# COVID-19 mortality risk for older men and women

**DOI:** 10.1186/s12889-020-09826-8

**Published:** 2020-11-19

**Authors:** N. David Yanez, Noel S. Weiss, Jacques-André Romand, Miriam M. Treggiari

**Affiliations:** 1 Department of Anesthesiology, 333 Cedar Street, TMP-3, New Haven, CT 06510 USA; 2 Department of Epidemiology, University of Washington, Seattle, Washington USA; 3 State Department of Health, Geneva, Switzerland

**Keywords:** Age, Severe acute respiratory syndrome-CoronaVirus-2, epidemiology, Pandemic, Population health

## Abstract

**Background:**

Case-fatality from COVID-19 has been reported to be relatively high in patients age 65 years or older. We sought to determine the age-specific rates of COVID-19 mortality at the population level.

**Methods:**

We obtained information regarding the total number of COVID-19 reported deaths for six consecutive weeks beginning at the 50th recorded death, among 16 countries that reported a relatively high number of COVID-19 cases as of April 12, 2020. We performed an ecological study to model COVID-19 mortality rates per week by age group (54 years or younger, 55–64 years, and 65 years or older) and sex using a Poisson mixed effects regression model.

**Results:**

Over the six-week period of data, there were 178,568 COVID-19 deaths from a total population of approximately 2.4 billion people. Age and sex were associated with COVID-19 mortality. Compared with individuals ages 54 years or younger, the incident rate ratio (IRR) was 8.1, indicating that the mortality rate of COVID-19 was 8.1 times higher (95%CI = 7.7, 8.5) among those 55 to 64 years, and more than 62 times higher (IRR = 62.1; 95%CI = 59.7, 64.7) among those ages 65 or older. Mortality rates from COVID-19 were 77% higher in men than in women (IRR = 1.77, 95%CI = 1.74, 1.79).

**Conclusions:**

In the 16 countries examined, persons age 65 years or older had strikingly higher COVID-19 mortality rates compared to younger individuals, and men had a higher risk of COVID-19 death than women.

## Background

After the emergence of a novel coronavirus (Severe Acute Respiratory Syndrome-CoronaVirus-2 [SARS-CoV-2]) in China, a pandemic has spread worldwide [1]. Clinical manifestations of SARS-CoV-2 infection have been designated as COVID-19. The epidemic that originated in the Hubei province in China spread to over 60 countries, with western Europe and the US being particularly severely affected by COVID-19.

Data from China and Italy suggest a case-fatality of 2.3% in patients with COVID-19, with more than 50% of the fatalities occurring in patients 50 years of age or older [2]. In the largest reported series from Northern Italy, case-fatality in patients 64 years or older was 36% compared with 15% in younger patients [3].

The mortality rate from COVID-19 in any age category is determined not only by the case-fatality among recognized incident cases, but by the age-specific incidence itself. Using age-specific mortality data, we sought to determine the relative risk of death across age categories in 16 countries that have had a high reported incidence of COVID-19 .

## Methods

Tabulations of COVID-19 deaths were obtained from the 21 countries with the highest recorded number of cases of COVID-19 as of April 12, 2020. Our data source for COVID-19 cases and deaths was the Johns Hopkins University, Center for Systems Science and Engineering Coronavirus Resource Center (CSSE). CSSE provides numbers of deaths and confirmed cases for each country across the globe [4]. The countries in our sample were (in alphabetical order): Austria, Belgium, Brazil, Canada, China, France, Germany, India, Iran, Israel, Italy, Netherlands, Portugal, Russia, South Korea, Spain, Sweden, Switzerland, Turkey, the United Kingdom, and the United States. We collected the total number of deaths each country over 6 weeks starting the day of a country’s fiftieth recorded COVID-19 death. On May 8, 2020, complete 6-weeks of data were available for 19 of the 21 countries. Age and sex distributions for COVID-19 deaths were procured for 16 of the 19 countries, predominantly from government ministries of public health. We estimated the number of COVID-19 deaths for each age and sex group for the 6-week totals of COVID-19 deaths for each country. COVID-19 mortality rates were determined using age and sex specific population sizes for each country using 2020 population estimates from the Central Intelligence Agency (CIA) World Factbook [5]. Table 1 provides variable definitions, all publicly available data sources, their provenance, and weblinks where these data can be extracted.

**Table 1:** Data Sources

Variables	Source	Data
COVID-19 Deaths	Johns Hopkins University the Center for Systems Science and Engineering (CSSE) Coronavirus Resource Center coronavirus.jhu.edu	Country level COVID-19 deaths up to May 8, 2020.
Age groups, sex, population sizes by age and sex	CIA World Factbookcia.gov/library/publications/the-world-factbook	Country-level population sizes (2020 est.) and demographic factors.
COVID-19 deaths by age and sex	info.gesundheitsministerium.at (AUT) info-coronavirus.be (BEL) covid.saude.gov.br (BRA) wikipedia.org/wiki/COVID-19_pandemic_in_Canada (CAN) qbitai.com/2020/02/11611.html (CHN) statista.com/statistics/1110092/coronavirus-covid-19-deaths-age-group-switzerland/ (CH) Robert Koch Institute (DEU) mscbs.gob.es/profesionales/saludPublica/ccayes/alertasActual/nCov-China/documentos/Actualizacion_96_COVID-19.pdf (ESP) santepubliquefrance.fr/maladies-et-traumatismes/maladies-et-infections-respiratoires/infection-a-coronavirus/documents/bulletin-national/covid-19-point-epidemiologique-du-15-mars-2020 (FRA) statista.com/statistics/1105061/coronavirus-deaths-by-region-in-italy (ITA) ncov.mohw.go.kr (KOR) rivm.nl/coronavirus-covid-19/grafieken (NLD) wikipedia.org/wiki/COVID-19_pandemic_in_Portugal (POR) statista.com/statistics/1107913/number-of-coronavirus-deaths-in-sweden-by-age-groups (SWE) www.ons.gov.uk/peoplepopulationandcommunity/birthsdeathsandmarriages/deaths/bulletins/deathsregisteredweeklyinenglandandwalesprovisional/weekending17april2020 (UK) www.cdc.gov/nchs/nvss/vsrr/covid_weekly (USA)	Joint distributions for BEL, ESP, DEU, KOR, NLD, POR, UK; marginal distributions for others.

### Statistical methods

We performed an ecological study to model the association between COVID-19 mortality as the outcome versus age and sex using a multilevel mixed-effects Poisson regression model. The predictor of particular interest, age, was categorized as: 0 to 54, 55 to 64 and 65 or older years. We modeled COVID-19 deaths (Deaths) as.

$$ \mathsf{\log}\left(\mathsf{E}\left[{\mathsf{Death}}_{\mathsf{i}\mathsf{jk}}\ |\ \mathsf{Age},\mathsf{Sex}\right]/{\mathsf{N}}_{\mathsf{i}\mathsf{jk}}\right)={\mathsf{b}}_{\mathsf{i}}+{\upbeta}_{\mathsf{0}}+{\upbeta}_{\mathsf{1}}\ {\mathsf{Age}}_{\left[\mathsf{55}-\mathsf{64}\right]}+{\upbeta}_{\mathsf{2}}\ {\mathsf{Age}}_{\left[\ge \mathsf{65}\right]}+{\upbeta}_{\mathsf{3}}\ {\mathsf{Sex}}_{\left[\mathsf{female}\right]} $$

where *E[Death*_*ijk*_ *| Age, Sex]* denotes the mean number of COVID-19 deaths by age (Age) and sex (Sex) groups. We incorporated a normal, mean zero country-level random effect, *b*_*i*_; to account for correlated data at the country level. The model indices denote the country (*i* = 1,2,…,16), age groups (*j* = 1 ages [≤ 54 years], 2 ages [55–64 years], 3 ages [≥ 65 years]) and sex (*k* = 1 for female, *k* = 0 for male); the country population sizes by age and gender groups, *N*_*ijk*_, were modeled as an offset. The exponentiated regression coefficients are interpreted as incidence rate ratios (IRR’s) of COVID-19 mortality. Six-week total COVID-19 deaths were disaggregated for each country using their respective estimated age and sex distributions of COVID-19 deaths. Our analyses accounted for this source of variation using a non-parametric bootstrap procedure. We selected 10,000 bootstrap samples from the estimated age and sex distributions of COVID-19 deaths to generate 10,000 disaggregated estimates of the COVID-19 death totals by age and sex for each country. Bootstrap standard errors were used in testing whether there were differences in the COVID-19 mortality rates between age groups and for sex. Specifically, Wald statistics were used to test for associations between the age groups and for sex. We provide 95% bootstrapped confidence intervals using the tolerance interval method on the 10,000 bootstrap estimates. Analyses were conducted using the Stata statistical package (StataCorp. 2019. Stata Statistical Software: Release 16. College Station, TX: StataCorp LP).

## Results

**Fig. 1:**
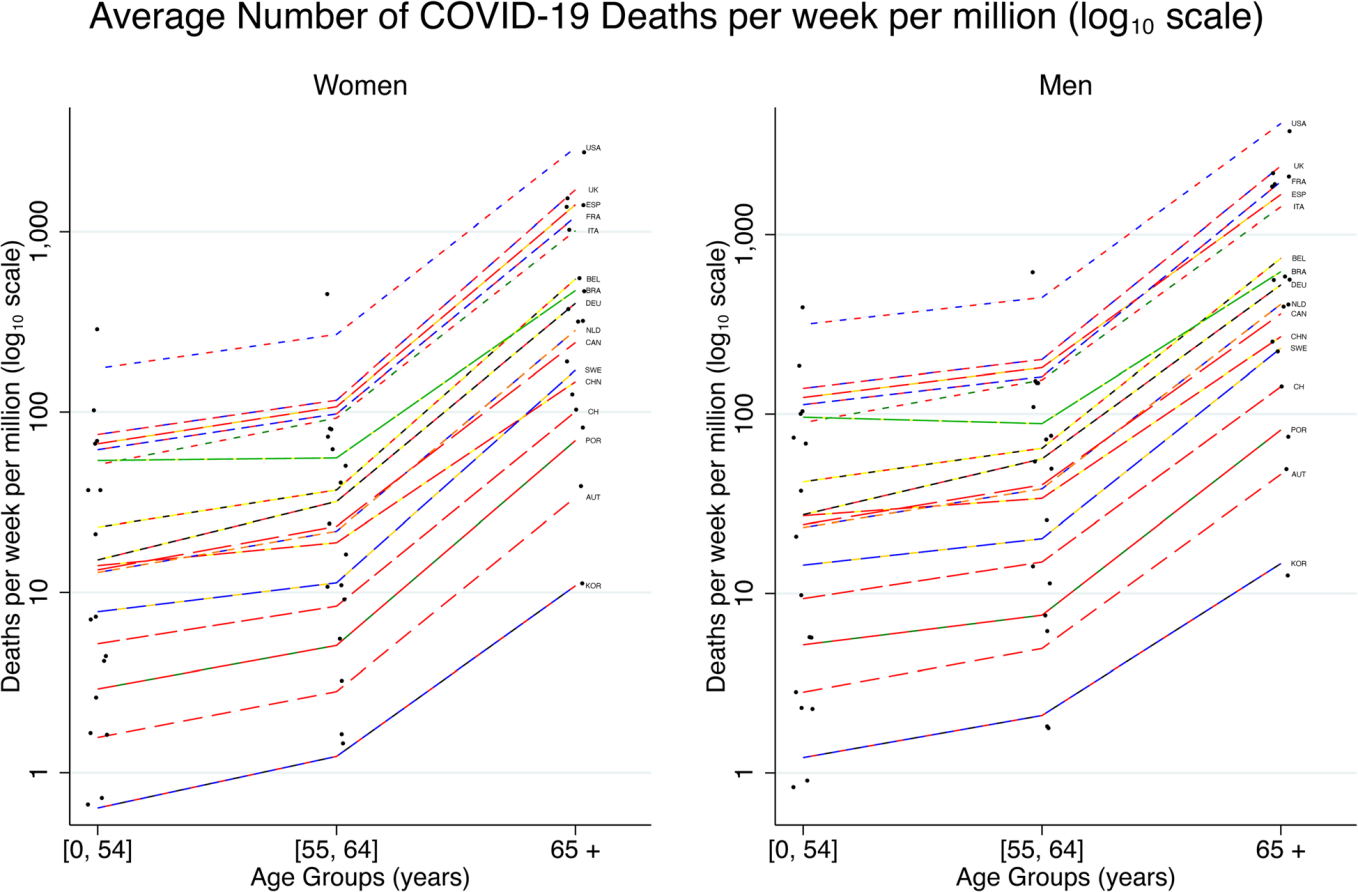
COVID-19 actual and estimated average number of weekly deaths for six consecutive weeks beginning with the 50th recorded death, among 16 countries that reported a relatively high number of COVID-19 cases as of April 12, 2020. Part A women, Part B men

**Fig. 2:**
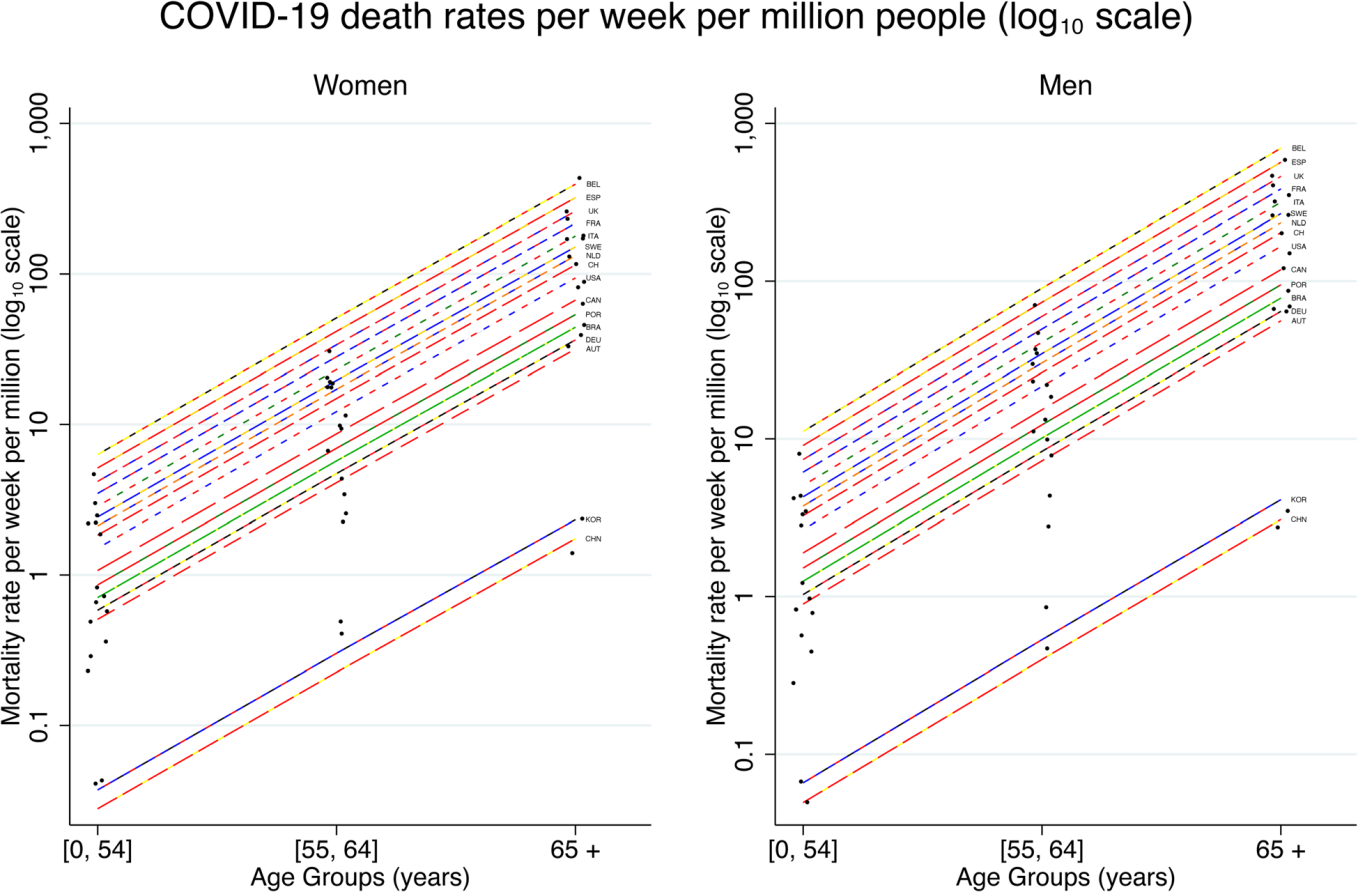
COVID-19 actual and estimated weekly death rates for six consecutive weeks beginning with the 50th recorded death, among 16 countries that reported a relatively high number of COVID-19 cases as of April 12, 2020. Part A women, Part B men

Of the 178,568 COVID-19 deaths reported in our six-week sample from a total population of approximately 2.4 billion people, 153,923 deaths (86.2%) were in persons age 65 years or older. Table 2 presents the cumulative number of COVID-19 deaths and COVID-19 mortality rates (per week per million persons) for the six specified age and sex categories. Figure 1 displays actual and estimated weekly number of COVID-19 deaths. The United States had the highest number of COVID-19 deaths per week, followed by several of the western European countries initially affected by COVID-19. Figure 2 shows actual and model estimated weekly averaged COVID-19 mortality rates for the three age groups, stratified by sex. We observed that eight of the 11 western European countries in our sample had the highest mortality rates, followed by the three countries in the Americas (US, Canada, Brazil) and then German and Austria. China and South Korea had the lowest COVID-19 mortality rates among the countries in our sample. By age groups, we see that the mortality rates and model estimates clearly show COVID-19 mortality rates have been higher in the older age categories.

**Table 2:** Total deaths and death rates per week per million stratified by age and sex

Country	Overall	Women			Men		
		0–54	55–64	65+	0–54	55–64	65+
Austria							
*Population (mil.)*	8.9	2.9	0.6	1.0	2.9	0.6	0.8
*Total deaths*	551	4	9	230	5	11	292
*Death rate*	10.4	0.2	2.5	38.3	0.3	3.1	60.8
Belgium							
*Population (mil.)*	11.7	3.9	0.8	1.3	4.0	0.8	1.0
*Total deaths*	7636	17	154	3453	15	348	3649
*Death rate*	108.8	0.7	32.1	442.7	0.6	72.5	608.2
Brazil							
*Population (mil.)*	212.0	85.4	10.9	11.2	86.1	9.8	8.3
*Total deaths*	7879	375	441	2336	562	661	3504
*Death rate*	6.2	0.7	6.7	34.8	1.1	11.2	70.4
Canada							
*Population (mil.)*	37.7	12.4	2.7	3.9	12.8	2.6	3.3
*Total deaths*	4487	25	53	1916	32	66	2395
*Death rate*	19.8	0.3	3.3	81.9	0.4	4.2	121.0
China							
*Population (mil.)*	1390	504.4	83.6	90.5	549.2	84.8	81.6
*Total deaths*	3016	137	233	716	243	414	1273
*Death rate*	0.4	0.0	0.5	1.3	0.1	0.8	2.6
France							
*Population (mil.)*	67.8	22.4	4.4	7.9	23.1	4.1	6.0
*Total deaths*	21,746	391	304	8003	587	457	12,004
*Death rate*	53.5	2.9	11.5	168.8	4.2	18.6	333.4
Germany							
*Population (mil.)*	80.2	24.3	6.3	10.3	24.9	6.3	8.1
*Total deaths*	6556	45	99	2719	132	302	3259
*Death rate*	13.6	0.3	2.6	44.0	0.9	8.0	67.1
Italy							
*Population (mil.)*	62.4	20.0	4.5	7.8	19.9	4.2	5.9
*Total deaths*	19,847	208	513	6225	386	953	11,562
*Death rate*	53.0	1.7	19.0	133.0	3.2	37.8	326.6
Korea, South							
*Population (mil.)*	51.8	17.1	4.1	4.7	18.5	3.9	3.6
*Total deaths*	182	4	9	73	5	10	82
*Death rate*	0.6	0.0	0.4	2.6	0.0	0.4	3.8
Netherlands							
*Population (mil.)*	17.3	5.7	1.2	1.9	5.8	1.2	1.6
*Total deaths*	4508	27	60	1867	37	140	2377
*Death rate*	43.4	0.8	8.3	163.8	1.1	19.4	247.6
Portugal							
*Population (mil.)*	10.3	3.4	0.7	1.3	3.4	0.6	0.9
*Total deaths*	1029	10	21	493	17	40	449
*Death rate*	16.7	0.5	5.0	63.2	0.8	11.1	83.1
Spain							
*Population (mil.)*	50.0	16.7	3.3	5.3	17.6	3.2	4.0
*Total deaths*	21,228	224	372	8215	449	890	11,078
*Death rate*	70.8	2.2	18.8	258.3	4.3	46.4	461.6
Sweden							
*Population (mil.)*	10.2	3.4	0.6	1.1	3.5	0.6	1.0
*Total deaths*	2792	39	66	1096	52	87	1453
*Death rate*	45.6	1.9	18.3	166.1	2.5	24.2	242.2
Switzerland							
*Population (mil.)*	8.4	2.8	0.6	0.9	2.9	0.6	0.7
*Total deaths*	1683	10	33	664	14	45	917
*Death rate*	33.4	0.6	9.2	123.0	0.8	12.5	218.3
United Kingdom							
*Population (mil.)*	65.8	22.1	4.2	6.7	23	4.1	5.5
*Total deaths*	23,990	592	477	8605	1071	899	12,345
*Death rate*	60.8	4.5	18.9	214.1	7.7	36.5	374.1
United States							
*Population (mil.)*	333.0	115.6	22.1	31.0	118.2	20.7	25.0
*Total deaths*	51,435	1783	2765	17,186	2436	3779	23,487
*Death rate*	25.7	2.6	20.9	92.4	3.4	30.4	156.6

We observed COVID-19 mortality rates for women in Fig. 2 (a) to be uniformly lower compared to the rates for men, Fig. 2 (b).

Estimates for our formal analysis compared COVID-19 mortality rates by age group and sex. We observed that individuals ages 55 to 64 years had 8.1 times higher COVID-19 mortality rate than individuals younger than 55 years of age (IRR = 8.1, 95% CI = 7.7, 8.5), and that those age 65 or older had a 62 times higher rate compared to the youngest group (IRR = 62.1, 95%CI = 59.7, 64.7). Persons age 65 or older had 7.7 times higher COVID-19 death rates than those between the ages of 55 and 64 years (IRR = 7.7, 95%CI = 7.4, 7.9). Finally, we observed that men had 1.77 times higher COVID-19 mortality rates than did women (IRR = 1.77, 95% CI = 1.74, 1.79).

## Discussion

In this study of COVID-19 in 16 countries, we found that COVID-19 mortality rates were strongly associated with older age and (to a lesser extent) with sex, with men having 77% higher mortality. Estimated IRRs were highest in the elderly. These observations suggest that the previously observed high COVID-19 case-fatality among older persons translates into a similarly high mortality rate at the population level.

Although case fatality rates have been initially reported to be similar across countries [2], they have been subsequently found to vary significantly among countries. Several factors may contribute to these differences including type of healthcare systems, patient characteristics, or prevalence of diagnostic testing [6]. Patient comorbidities such as hypertension, diabetes, and obesity have been shown to be associated with higher COVID-19 mortality [7]. Since the number of comorbid conditions steadily increases with age, this could be another logical explanation of the observed increased mortality in older patients. While disease mortality is higher in the elderly in other conditions like cardiovascular disease, changes associated with immunsenscence might explain the increased vulnerability to infection and the disproportionately high mortality due to COVID-19 in older patients [6].

Consistent with our findings, in a recent meta-analysis of all-cause mortality from four countries (China, Italy, Spain, United Kingdom), and New York State, the percentage of octogenarians was found to be different across the regions with the lowest cohort being in China and the highest in New York State. Similarly, the lowest mortality was observed in China (3.1%) and the highest in New York State (21%) [8]. The largest increase in mortality risk was observed in patients aged 60 to 69 years. Therefore, it is important to report age-specific rates of COVID-19 mortality that account for these sources of variability in the population.

While determining the incidence of COVID-19 in a given population depends in part on the prevalence and quality of testing for the presence of this infection, reporting of mortality should be less affected by these factors. Nonetheless, COVID-19 deaths will have been underreported to the extent that persons may die without being identified as positive for SARS CoV-2. To the extent that this underascertainment of COVID-19 mortality is relatively greater in the elderly, we would have underestimated the magnitude of the relative mortality rate among persons in this age group. However, we recognize that the major limitations of this study is related to the possible differences in reporting of attributable deaths across countries, which would affect the rates reported by each country. Another limitation is the lack of available data on confounding factors e.g. ethnicity and patient co-morbidities known to be risk factors for COVID-19 mortality [9–11].

## Conclusions

As policy-makers prepare to make decisions on how to curb the outbreak, while reducing the pressures on the healthcare system and reopening the economy, it will be important that future choices be tailored to account for the demographics of the population and specifically consider the prevalence of people ager 65 or older in the population in specific regions [12], or communities in which nursing homes are located [13, 14]. Within countries, mapping of regional age distribution potentially could help identify areas at particularly high risk of being affected [15]. At a more granular level, tracking older population dynamics and interactions may provide further guidance on how to protect the more vulnerable older population.

## Data Availability

Datasets compiled for this study are publicly available. Specific data sources are detailed in the Methods section and Table 1 of the manuscript.

## Abbreviations

SARS-CoV-2: Severe Acute Respiratory Syndrome-CoronaVirus-2
IRR: Incidence Rate Ratio
CI: Confidence Interval
CSSE: Center for Systems Science and Engineering
CIA: Central Intelligence Agency
